# Supplementary material for: Nucleation and growth of orbital ordering
Source: Nat Commun. 2020 May 11;11:2324. doi: 10.1038/s41467-020-16004-2 (PMC7214450; doi:10.1038/s41467-020-16004-2)
Supplement: Supplementary file 1 — Supplementary information [file 41467_2020_16004_MOESM1_ESM.pdf]

Supplementary Information for

Nucelation and growth of orbital ordering

Katsufuji *et al.*

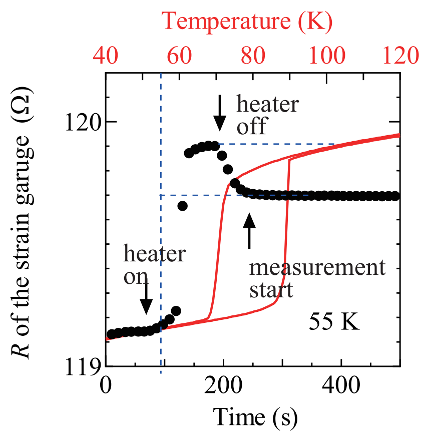

Supplementary Figure 1: A typical time dependence of the process for the strain measurement (at 55 K) after rapid cooling (the black circles, lower axis) and the temperature dependence of the strain (the red lines, upper axis)

Supplementary Methods 1: Details of the experiment with rapid cooling

As discussed in the methods section in the main text, for rapid cooling in the resistivity and strain measurement, a heater was attached to the sample, and the temperature of the cryostat is kept at a specific temperature and then, an appropriate magnitude of electric power was applied to the heater so that the temperature of the sample locally increases. Then, we turned off the electric power to the heater and waited until the value of the resistivity or the strain becomes stable. After that, we started measuring the time dependence. A typical example of the time dependent process is shown in Supplementary Fig. 1.

Supplementary Note 1: Summary of the physical properties of $BaV_{10}O_{15}$

Physical properties of $BaV_{10}O_{15}$ and a related compound are summarized here (Refs. [33, 34] in the main text and Supplementary Refs. [1, 2].)

In $BaV_{10}O_{15}$, V ions form a quasi-triangular lattice along the *ab* plane.

Namely, a V triangular lattice from which V triangles are periodically missing leads to a lattice in which "V boats", each of which are composed of five 5 V ions, are aligned in a centered orthorhombic lattice. The bilayer stacking of these lattices along the c axis is a building block of $BaV_{10}O_{15}$ (Fig. 1**a** of the main text). The average valence of the V ions is 2.8+ ($3d^{2.2}$. Nominally, four out of five V ions are 3+ ($3d^2$) and remaining one V ion is 2+ ($3d^{3+}$).

$BaV_{10}O_{15}$ exhibits a structural phase transition (from the space group *Cmce* to *Pbca*) at $T_c \sim 130$ K. This structural phase transition is characterized by a formation of V trimers over the bilayer, in which the V-V bonds are shortened by several percent below T_c (Fig. 1**b**). With this V trimerization, three out of five V ions form trimers whereas two V ions remain lone V ions. According to a measurement of resonance x-ray scattering (Ref. [34]), this structural phase transition is caused by the orbital ordering of the V t_{2g} states; namely, at each side of the V triangle, either xy, yz, or zx orbitals of neighboring V ions, which are supposed to be 3+ ($3d^2$), form a spin-singlet bond. Upon this phase transition, the b lattice constant decreases by $\sim 0.6\%$. whereas the a and c lattice constants increase by $\sim 0.2\%$.

Associated with this structural phase transition in $BaV_{10}O_{15}$, the electrical resistivity jumps by three orders of magnitude. Furthermore, a charge gap opens with the phase transition, as seen in the optical conductivity spectrum (Ref. [33]) and the photoemission spectrum (Supplementary Ref. [2]). The magnetic susceptibility gradually increase with decreasing T above T_c, indicating a Curie-Weiss behavior with a Weiss constant of 1.2×10^3 K, and at T_c, the magnetic susceptibility slightly jumps (increases). This increase in the magnetic susceptibility in spite of the formation of a spin-singlet state in the V trimers is caused by the magnetic moments of the lone V ions. With further decreasing T, the magnetic moments on the lone V ions order antiferromagnetically at ~ 30 K, resulting in the cusp in the magnetic susceptibility along the a axis.

It is known that $SrV_{10}O_{15}$, which has the same crystal structure as $BaV_{10}O_{15}$, does not exhibits any structural phase transition down to the lowest temperature (Supplementary Ref. [1]).

Supplementary Note 2: Magnetic susceptibility measured with various sweep rates of the temperature

Supplementary Figure 2 shows the magnetic susceptibility χ_a of $BaV_{10-x}Ti_xO_{15}$

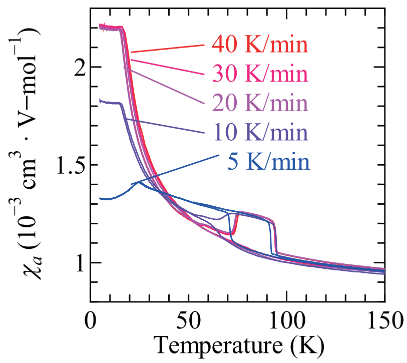

Supplementary Figure 2: Magnetic susceptibility χ of $BaV_{10-x}Ti_xO_{15}$ with $x = 0.15$ measured in the cooling run with various sweep rates of the temperature from 300 K and 5 K and in the warming run with the rate of 5 K/min from 5 K to 300 K.

with $x = 0.15$ measured in the cooling run with various sweep rates of the temperature from 300 K and 5 K and in the warming run with the rate of 5 K/min from 5 K to 300 K. As can be seen, the behaviors are almost the same for χ with the rates higher than 20 K/min, but they are different with the rates lower than 10 K/min. This experimental result indicates that if the sweep rate of the temperature in the cooling run is higher than 20 K/min, a supercooled state can be obtained in this sample.

Supplementary Note 3: Time dependence of the strain for $Ba_{1-x}Sr_xV_{10}O_{15}$

In the main text, we discuss $BaV_{10}O_{15}$ of which the transition temperature is decreased by the Ti doping to the V site. Another way of reducing the transition temperature is Sr doping to the Ba site. Supplementary Figure 3 shows the time dependence of the strain $\Delta L/L$ after rapid cooling (30 K/min) from $T > 100$ K to various temperatures for $Ba_{1-x}Sr_xV_{10}O_{15}$ with $x = 0.06$. As can be seen, the time dependence of $\Delta L/L$ and its temperature dependence are similar to those of $BaV_{10-x}Ti_xO_{15}$ with $x = 0.15$ shown in Fig. 3**c** of the main text, though the time dependence between 58 and 75 K cannot be measured because of the too small transformation time τ in this temperature range for the present compound. This indicates that the nucleation-growth behavior discussed in the main text can be observed

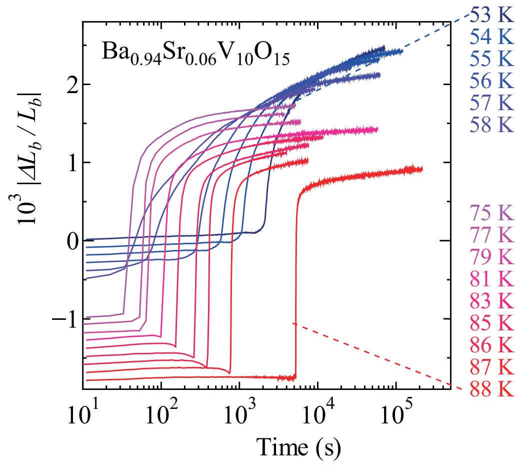

Supplementary Figure 3: The time dependences of the strain $\Delta L/L$ after rapid cooling (30 K/min) from $T > 100$ K to various temperatures for $Ba_{1-x}Sr_xV_{10}O_{15}$ with $x = 0.06$.

irrespective of the way of doping.

Supplementary Note 4: Relation between the strain and the volume fraction in composite materials

In the case of the two-phase coexistence, we need to consider the strain of the materials with the mixture of two phases, A and B, as composite materials. Here, we assume that the A and B phases are both single crystals with the orthorhombic structure, and the a, b, and c axes of the A and the B phases are parallel to each other in the material. We consider the compressive or tensile strain measured by a strain gauge for such a material.

We assume that the material is composed of $N = N_1 \times N_2 \times N_3$ rectangular parallelepipeds whose edges are parallel to the x, the y, and the z axis, and each of the parallelepipeds are either in the A phase or the B phase. The ratio of the A phase in the material is assumed as x $(0 \leq x \leq 1)$. This means that among the $N = N_1N_2N_3$ parallelepipeds, xN are in the A phase and $(1-x)N$ are in the B phase, and they are randomly distributed in a three-dimensional manner in the material (Supplementary Fig. 4 (a)).

Here, we ignore the bulk modulus of both the A and B phases. We also

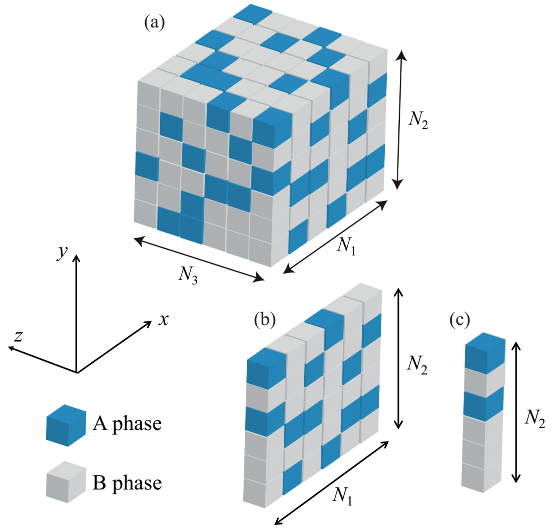

Supplementary Figure 4: The material composed of $N = N_1 \times N_2 \times N_3$ rectangular parallelepipeds, each of which are either in the A phase or the B phase.

assume that a strain gauge is attached to the plane normal to the z axis. This plane is composed of the $N_1 \times N_2$ parallelepipeds at $z = z_i$ ($i = 1 \sim N_3$) of $i = 1$. Among the $N_1 \times N_2$parallelepipeds, xN_1N_2 of them are in the A phase and $(1-x)N_1N_2$ of them are in the B phase (Supplementary Fig. 4 (b)). When a strain gauge is attached to the plane, the gauge measures the strain along, for example, the y axis. This means that the strain gauge measures the length of the line along the y axis that is composed of N_2 parallelepipeds at, for example, $x = x_j$ ($j = 1 \sim N_1$) of $j = 1$. Among the N_2 parallelepipeds, xN_2 are in the A phase and $(1-x)N_2$ are in the B phase (Supplementary Fig. 4 (c)). If we assume that the b lattice constants for the A and B phases are b_A and b_B, respectively, and α is defined as $\alpha = b_A/b_B - 1$, and the elastic energy in the material is ignored, the strain $\Delta L/L$ along the b axis associated with the appearance of the A phase becomes $x\alpha$. Thus, the strain is proportional to the volume fraction of the A phase in the material, x.

For the treatment of the case when the elastic energy cannot be ignored, see Supplementary Ref. [3].

Supplementary Note 5: Fitting of the time-dependent strain

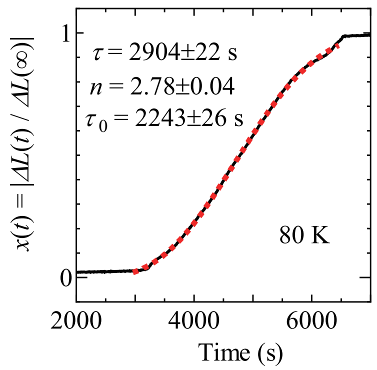

Supplementary Figure 5: Experimental results of the time-dependent strain (the solid line) and the fitting curve (the dashed line).

Supplementary Figure 5 shows the experimental results of $x(t) = |\Delta L(t)/\Delta L(\infty)|$ (the solid line) at 80 K and the fitting by the functional form of $x(t) = 1 - \exp\left\{-\left(\frac{t-\tau_0}{\tau}\right)^n\right\}$ (the dashed line) with fitting parameters of τ (transformation time), τ_0 (transient time), and n (Avrami exponent). As can be seen, the experimental result can be fitted satisfactorily, and three parameters can be obtained with good accuracies.

Supplementary Note 6: Time dependence dominated by the diffusive motion of domain boundaries

Supplementary Figure 6 shows the plot of $\ln x(t) = \ln|\Delta L(t)/\Delta L(\infty)|$ vs. $\ln t$ below 69 K. As can be seen, the slope of the initial increase is $\sim 1/2$ at all the temperatures, indicating the relation of $x(t) = \beta t^{1/2}$ in this t range. Such a $t^{1/2}$ dependence of the physical properties, which should be attributed to a diffusive motion of the domain boundary, has been observed in a perovskite manganite. As shown in Fig. 4**f** of the main text, for the phase transition of the present crystal, a more than 80 % volume fraction exhibits a nucleation-growth behavior and only a less than 20 % volume fraction exhibits a diffusive motion. Furthermore, the volume fraction of the diffusive motion decreases with increasing T. We speculate that near the defect or near the surface of the crystal, the nucleation-growth behavior is suppressed because of the strain, which induces a diffusive motion of the domain boundary, particularly at low temperatures.

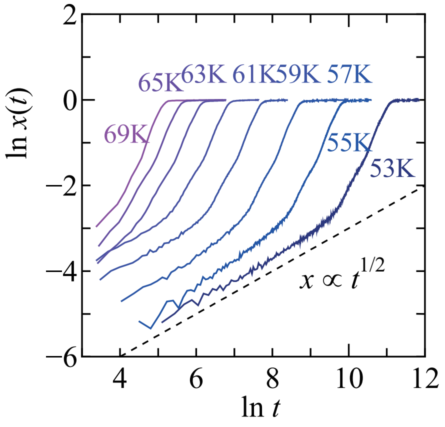

Supplementary Figure 6: $\ln x(t)$ vs $\ln t$, where $x(t) = |\Delta L(t)/\Delta L(\infty)|$

Supplementary Note 7: Time dependence of other quantities

Supplementary Figure 7 (a) shows the Avrami plot of the resistivity ρ. Namely, $\ln(-\ln(1-x_\rho(t)))$ with $x_\rho(t) = (\rho(t)-\rho(0))/(\rho(\infty)-\rho(0))$ is plotted as a function of $\ln t$. As can be seen, $\ln t$ dependence of the data is not monotonic, but there is a two-step structure. In addition, there is a small decrease in $\ln(-\ln(1-x_\rho(t)))$ before it starts to increase, as shown Supplementary Fig. 7 (a).

Resistivity is not a bulk sensitive quantity and thus, we calculate the resistivity from the volume fraction of the insulating domains estimated from the strain $\Delta L/L$ by the effective-medium approximation (EMA) (Ref. [24] in the main text), and compare the result with the one experimentally obtained, as show in Supplementary Fig. 7 (b). As can be seen, such a nonmonotonic structure cannot be reproduced by EMA.

It is likely that the time dependence of the resistivity experimentally obtained is partly dominated by the inhomogeneity of the sample. Note that the strain measurement by the strain gauge is dominated mainly by the region near the strain gauge in the sample, but the resistivity measurement is dominated by the whole region of the sample and thus, is subject to inhomogeneity more. In addition, there is anisotropy in the resistivity for the present compound, and accordingly, inhomogeneity affects how the electric current flows in the sample. This can lead to a complicated behavior of the resistance, including the two-step structure and small decrease, associated

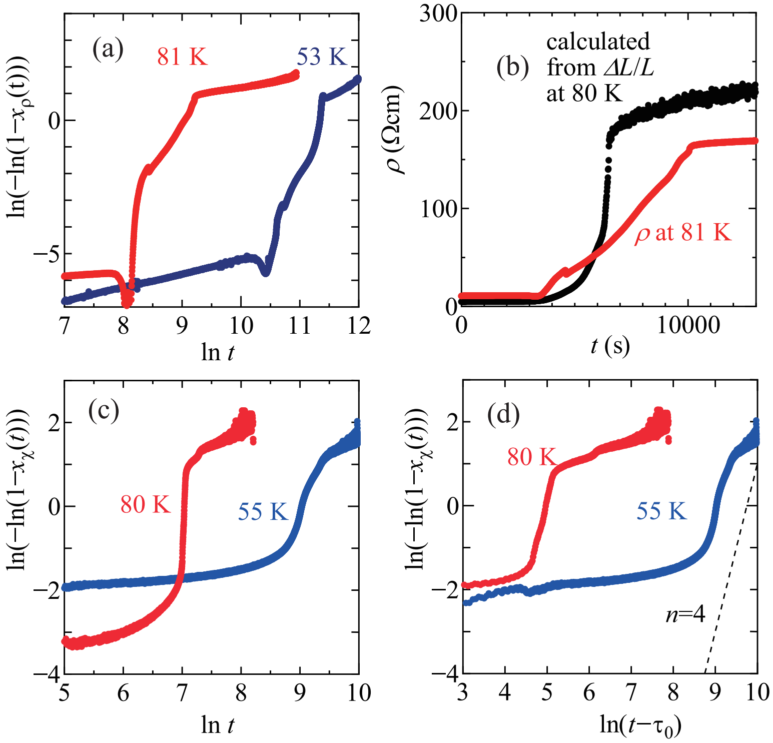

Supplementary Figure 7: (a) Avrami plot for the resistivity. (b) Comparison between the experimentally obtained resistivity and the calculated resistivity by the effective-media approximation from the experimentally obtained strain $\Delta L/L$. (c) Avrami plot for the magnetic susceptibility. (d) The same as that in (c) except that the x axis is $\ln(t - \tau_0)$ with $\tau_0 = 1 \times 10^3$ s for the data at 80 K.

with the nucleation-growth behavior.

Supplementary Figure 7 (c) shows the Avrami plot of the magnetic susceptibility χ; namely, $\ln(-\ln(1-x_\chi(t)))$ with $x_\chi(t) = (\chi(t)-\chi(0))/(\chi(\infty)-\chi(0))$ is plotted as a function of $\ln t$. Unlike the case of the resistivity, $\ln t$ dependence of the data from the magnetic susceptibility is qualitatively similar to the behavior of the strain discussed in the main text. Namely, the slope of $\ln(-\ln(1-x_\chi(t)))$ vs $\ln t$ near T_c (80 K) is larger than four, whereas that at low T (55 K) is close to four. In this case, however, we cannot uniquely obtain τ, τ_0, and n by the fitting of $x_\chi(t) = 1-\exp\left\{-\left(\frac{t-\tau_0}{\tau}\right)^n\right\}$, unlike the case of the strain. Supplementary Figure 7 (d) shows a plot of $\ln(-\ln(1-x_\chi(t)))$ as a function of $\ln(t-\tau_0)$, where τ_0 is determined quite arbitrarily.

Supplementary Note 8: Fitting of the time-temperature-transformation curve

Supplementary Figure 8 shows the experimental results of the transformation time τ vs T (solid circles) and the fitting by Eq. (4) in the main text (the black line), together with the curves of $\tau = \tau_0 \exp(T_1/T)$ (the blue line), $\tau = \tau_0 \exp(T^3/(T_\mathrm{c}-T)^2 T)$ (the red line), and $\tau = \tau_0$ (the dashed line) with $T_1 = 2133$ K, $T_2 = 87.6$ K, $T_\mathrm{c} = 103$ K, and $\tau_0 = 1.0 \times 10^{-15}$ s. As can be seen, the term $\exp(T_1/T)$ with $T_1 = 2133$ K is responsible for the increase in τ at lower temperatures whereas the term $\exp(T_2^3/(T_\mathrm{c}-T)^2 T)$ with $T_2 = 87.6$ K and $T_\mathrm{c} = 103$ K is responsible for the increase at high temperatures near the transition temperature. Note that τ is proportional to $(N_0 v_0^3)^{-1/4}$, where N_0 is the nucleation rate and v_0 is the linear speed of the nucleus, and N_0 is proportional to $\exp(T_1/T)\exp(T_2^3/(T_\mathrm{c}-T)^2 T)$ whereas v_0 is proportional to $\exp(T_1/T)$. Therefore, the increase in τ with decreasing T is dominated by the decrease in the speed of the nuclear growth, whereas the increase in τ near the transition temperature is dominated by the decrease in the nucleation rate.

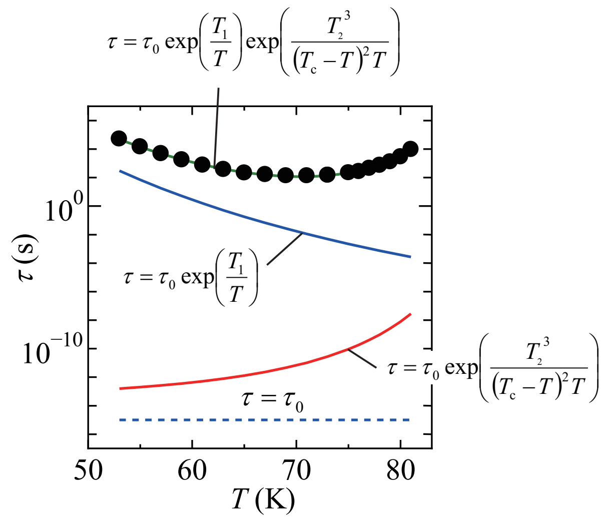

Supplementary Figure 8: Experimental results of the transformation time τ (the solid circles), the fitting curve (the black line), and the components of the fitting function (the blue line, red line, and dashed line).

Supplementary Discussion 1: Time dependence in the volume fraction of the transformed phase

Let us discuss the first-order phase transition from an α phase to a β phase dominated by a nucleation-growth process (Ref. [7, 10, 39, 40] in the main text). In such a case, in the sea of the α phase, nuclei of the β phase appear randomly with a specific rate, and once the nuclei appear, they continuously grow in size by a specific speed until all the system is occupied by the grown nuclei of the β phase. More specifically, a small cluster of the β phase (called embryo) does not continuously grow but its size is fluctuating until it reaches a critical size of the cluster, but once it does, the nucleus continuously grows in size (Supplementary Fig. 9).

First, how the time (t) dependence of the volume fraction of the transformed phase (the volume of the β phase divided by the total volume), $V(t)$, is modified when the impingement of the grown nuclei is taken into account. We define a real volume fraction of the transformed phase $V(t)$, and an "extend" volume fraction of the transformed phase $V_{\mathrm{ex}}(t)$, for which the growth of a nucleus in the already transformed region (a "phantom" region) is also counted (*i.e.*, the overlap of the nuclei is allowed). With this assumption, the

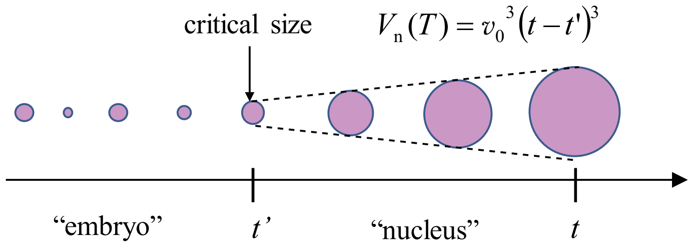

Supplementary Figure 9: Schematic pictures of an embryo and a nucleus.

increase in $V_{ex}(t)$, dV_{ex}, can always occur but the increase in $V(t)$, dV, occurs only when the growth occurs in the untransformed region (Supplementary Fig. 10). This leads to the following relation between the two quantities:

$$dV = (1 - V)dV_{ex}. \tag{1}$$

By integrating Eq. (1) with respect to t, and by considering the fact that $V = 0$ when $V_{ex} = 0$, we can obtain the following relation:

$$V(t) = 1 - \exp\left(-V_{ex}(t)\right). \tag{2}$$

Next, let us consider how to calculate $V_{ex}(t)$, the "extended" volume fraction of the transformed phase, based on the nucleation-growth process. Since the overlap of the nuclei is allowed for $V_{ex}(t)$, it is given by

$$V_{ex}(t) = \int_0^t V_n(t - t')N(t')dt', \tag{3}$$

where $N(t')$ is the nucleation rate (the number of nuclei that are born over a unit time) at t' per unit volume and $V_n(t - t')$ is the volume of a nucleus at t that was born at t' (Supplementary Fig. 9). The integral with respect to t' is taken because nuclei can be born at any time from 0 to t.

Here, we assume that nuclei grow isotropically in the three-dimensional space and the growth speed is dominated by a constant speed of the interface, v_0. With this assumption, the volume $V_n(t)$ is given by $V_n(t) = v_0^3 t^3$. If we assume that the nucleation rate does not depend on t but is a constant,

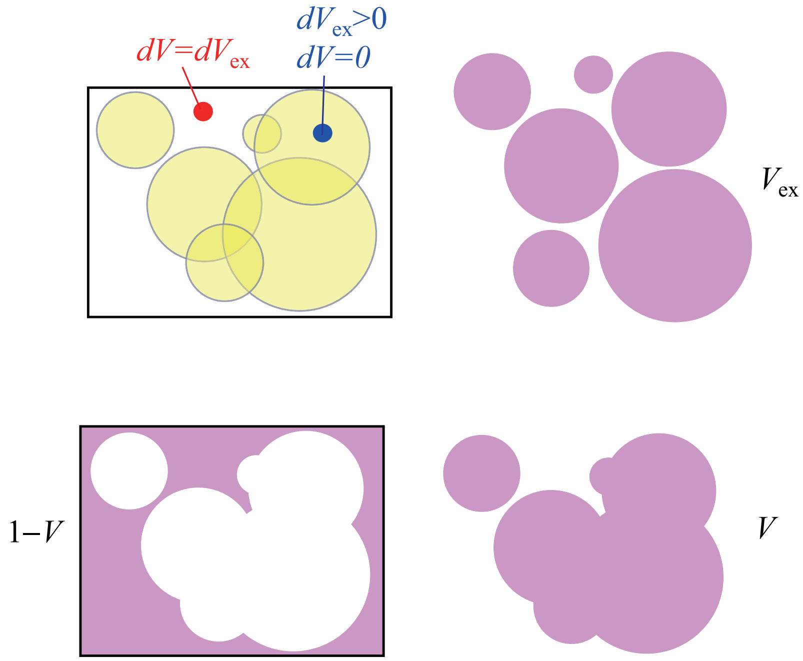

Supplementary Figure 10: Schematic pictures of the real volume fraction $V(t)$ and the extended volume fraction $V_{\mathrm{ex}}(t)$.

$N(t) = N_0$, we can calculate the integral of Eq. (3)

$$\begin{aligned} V_{\rm ex}(t) &= \int_0^t N_0 v_0^3 (t-t')^3 dt' \\ &= \frac{N_0 v_0^3}{4} t^4. \end{aligned} \tag{4}$$

Thus, the real volume fraction is given by

$$\begin{aligned} V(t) &= 1 - \exp\left(-\frac{N_0 v_0^3}{4} t^4\right) \\ &= 1 - \exp\left(-\left(\frac{t}{\tau}\right)^4\right), \end{aligned} \tag{5}$$

where the transformation time τ is given by

$$\tau = \left(\frac{4}{N_0 v_0^3}\right)^{1/4}. \tag{6}$$

In reality, however, the nucleation rate depends on time because immediately after the rapid cooling to a certain temperature, there is no nucleus ($N(t=0)=0$) or even no embryo. Thus, it takes some time until the distribution of the size of various clusters becomes in a steady state and $N(t)$ becomes almost time-independent. The functional form of $N(t)$ has been calculated theoretically, but for the analysis of the experimental data, we assume a step function for $N(t)$, namely, $N(t) = 0$ for $t < \tau_0$ and $N(t) = N_0$ for $t \geq \tau_0$, where τ_0 is the transient time. Substituting this $N(t)$ into Eq. (5), we find

$$V(t) = 1 - \exp\left(-\left(\frac{t-\tau_0}{\tau}\right)^4\right), \tag{7}$$

where τ is the same as Eq. (6). Note the difference between τ (transformation time) and τ_0 (transient time).

Supplementary Discussion 2: Temperature dependence of the parameters and the TTT curve

In this section, we discuss the temperature (T) dependence of the parameters, N_0 and v_0, and also the time-temperature-transformation (TTT) curve.

We assume that the difference of the Gibb's free energy per unit volume between the α phase and the β phase (the energy of the β phase is lower) is $\Delta g > 0$, and the interfacial energy per unit area between the α phase and the β phase is $\sigma > 0$. With these quantities, the free energy of a cluster of the β phase in a sphere shape with a radius of r is given by

$$\Delta G_n = -\frac{4}{3}\pi r^3 \Delta g + 4\pi r^2 \sigma. \tag{8}$$

With increasing r, ΔG_n first increases and takes a maximum at

$$r^* = \frac{2\sigma}{\Delta g}, \tag{9}$$

and then, it decreases with further increasing r (Supplementary Fig. 11). This r^* corresponds to the critical size of a cluster and if $r > r^*$, the cluster becomes a nucleus and starts growing. If $r < r^*$, the cluster is called an embryo and its size is thermally fluctuating. ΔG_n at $r = r^*$ is

$$\Delta G^* = \frac{16\pi\sigma^3}{3(\Delta g)^2}, \tag{10}$$

and this corresponds to a barrier height to overcome to change the fluctuating cluster (embryo) into a growing nucleus.

Based on this discussion, the nucleation rate N_0 is given by

$$N_0 = A \exp\left(-\frac{\Delta G_a}{k_B T}\right) \exp\left(-\frac{\Delta G^*}{k_B T}\right). \tag{11}$$

Here, A is approximately given by $A \sim n\nu_{\mathrm{ph}}$, where ν_{ph} is the characteristic frequency of the motion occurring when an atom is added to the ordered state (approximately, frequency of a phonon) and n is the number of atoms per unit volume. ΔG_a is the height of the energy barrier when an atom is added to the ordered state (Supplementary Fig. 12). Note the difference between this barrier height ΔG_a (barrier for an atom) and the barrier height ΔG^* discussed above (barrier for a cluster).

On the other hand, the speed of the interface v_0 is given by

$$v_0 = B \exp\left(-\frac{\Delta G_a}{k_B T}\right), \tag{12}$$

where B is approximately given by $B \sim a\nu_0$, and $a = n^{-1/3}$ is a distance between atoms. If we take account of the reverse transformation (from the

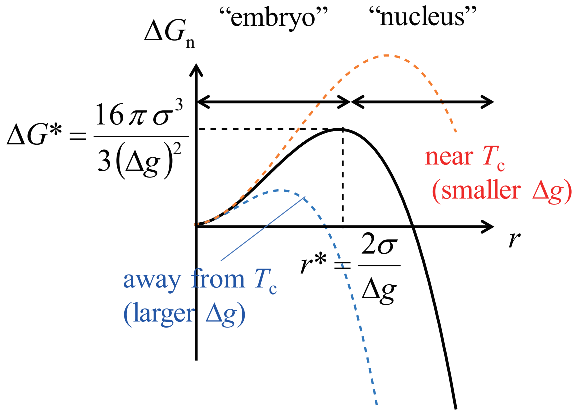

Supplementary Figure 11: Size (r) dependence of the free energy for a cluster of the β phase in the sea of the α phase with a different bulk free energy and the interfacial energy in between.

β phase to the α phase), the factor $(1 - \exp(-\Delta g/nk_BT)) \sim \Delta g/nk_BT$ should appear, but this can be safely ignored since the T dependence of $\exp(-\Delta G_a/k_BT)$ is by far dominant.

By substituting Eq. (10) into Eq. (11) and by substituting Eqs. (11) and (12) into Eq. (6), we find

$$\tau = \frac{\sqrt{2}}{\nu_{\mathrm{ph}}} \exp\left(\frac{\Delta G_a}{k_BT}\right) \exp\left(\frac{4\pi\sigma^3}{3(\Delta g)^2 k_BT}\right). \tag{13}$$

Now, let us assume the T dependence of Δg. If we ignore the difference

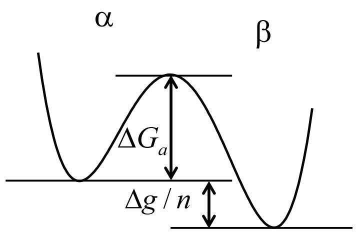

Supplementary Figure 12: Schematic picture for the free energy of the α phase and the β phase with an energy barrier in between.

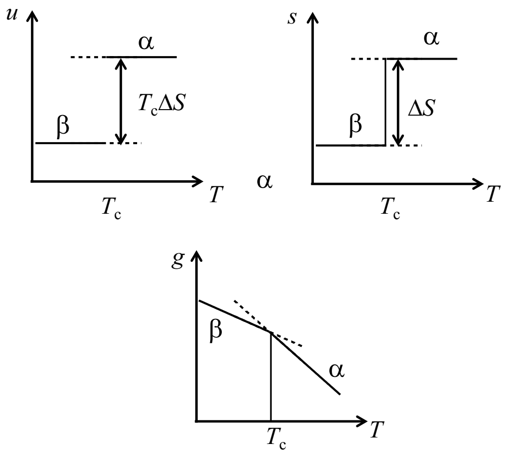

Supplementary Figure 13: Assumed internal energy u, entropy s, and free energy g per unit volume as a function of temperature in the α phase and the β phase.

in specific heat between the two phases but only take account of the latent heat at the phase transition, then

$$\Delta g = \Delta s(T_c - T), \tag{14}$$

where Δs is the jump of entropy per unit volume at the phase transition, and T_c is the (hypothetical) transition temperature, where the free energy of the α phase and the β phase becomes the same. Note that $T_c\Delta s$ is the latent heat per unit volume for the phase transition (Supplementary Fig. 13). The phase transition in the present compound is dominated by the orbital ordering of the V ions and considering the number of degeneracy in the t_{2g} states, Δs is $n(\ln 3)k_B$. Here, we assume

$$\Delta s = n\eta k_B, \tag{15}$$

where η is a number close to unity. For σ, we define an "interfacial energy per atom", I, and with this quantity, σ is given by

$$\sigma \sim Ia^{-2} = In^{2/3}. \tag{16}$$

By substituting Eqs. (14)(15(16) into Eq. (13), we obtain

$$\tau = \frac{\sqrt{2}}{\nu_{\rm ph}} \exp\left(\frac{\Delta G_a}{k_B T}\right) \exp\left(\frac{4\pi}{3\eta^2} \frac{I^3}{k_B^3 (T_{\rm c} - T)^2 T}\right). \tag{17}$$

This means that the TTT curve is given by

$$\tau = \tau_{\rm ph} \exp\left(\frac{T_1}{T}\right) \exp\left(\frac{T_2^3}{(T_{\rm c} - T)^2 T}\right). \tag{18}$$

Here, T_1 corresponds to the height of the energy barrier in adding one atom to the ordered phase (probably caused by the lattice distortion associated with the phase transition). T_2 corresponds to the height of the energy barrier to overcome when a cluster of the ordered phase becomes a growing nucleus, corresponding to the interfacial energy between the α phase and the β phase. $\tau_{\rm ph}$ is the inverse frequency of the characteristic phonon for the phase transition.

Supplementary References

[1] M. Hoshino, T. Kajita, T. Kanzaki, M. Uchida, Y. Tokura, and T. Katsufuji. *Phys. Rev. B*, 85:085106, 2012.

[2] T. Yoshino et al. *Phys. Rev. B*, 95:075151, 2017.

[3] R. A. Schapery. *J. Compos. Mater.*, 2:380, 1968.
